# Born in Bradford’s Better Start (BiBBS) interventional birth cohort study: Interim cohort profile

**DOI:** 10.12688/wellcomeopenres.18394.2

**Published:** 2023-09-21

**Authors:** Josie Dickerson, Sally Bridges, Kathryn Willan, Brian Kelly, Rachael H. Moss, Jennie Lister, Chandani Netkitsing, Amy L. Atkinson, Philippa K. Bird, Eleanora P. Uphoff, Dan Mason, Alex Newsham, Dagmar Waiblinger, Rifat Razaq, Sara Ahern, Maria Bryant, Sarah L. Blower, Kate E. Pickett, Rosemary M. McEachan, John Wright

**Affiliations:** 1 Born in Bradford, Bradford Teaching Hospitals National Health Service Foundation Trust, Bradford, BD9 6RJ, UK; 2 Health Sciences, University of York, York, YO10 5DD, UK; 3 Hull York Medical School, University of York, York, YO10 5DD, UK

**Keywords:** Interventional cohort, birth cohort, early years interventions, trials within cohorts, pragmatic randomised controlled trials, quasi-experimental designs, ethnic minority, deprivation

## Abstract

**Background:** The Born in Bradford’s Better Start (BiBBS) interventional birth cohort study was designed as an innovative cohort platform for efficient evaluation of early life interventions delivered through the Better Start Bradford programme. There are a growing number of interventional cohorts being implemented internationally. This paper provides an interim analysis of BiBBS in order to share learning about the feasibility and value of this method.

**Methods:** Recruitment began in January 2016 and will complete in December 2023 with a target sample of 5,000 pregnancies. An interim analysis was completed for all pregnancies recruited between January 2016 and November 2019 with an expected due date between 1 ^st^ April 2016 and 8 ^th^ March 2020. Descriptive statistics were completed on the data.

**Results:** Of 4,823 eligible pregnancies, 2,626 (54%) pregnancies were recruited, resulting in 2,392 mothers and 2,501 children. The sample are representative of the pregnant population (61% Pakistani heritage; 12% White British; 8% other South Asian and 6% Central and Eastern European ethnicity). The majority of participants (84%) live in the lowest decile of the Index of Multiple Deprivation, and many live in vulnerable circumstances. A high proportion (85%) of BiBBS families have engaged in one or more of the Better Start Bradford interventions. Levels of participation varied by the characteristics of the interventions, such as the requirement for active participation and the length of commitment to a programme.

**Conclusions:** We have demonstrated the feasibility of recruiting an interventional cohort that includes seldom heard families from ethnic minority and deprived backgrounds. The high level of uptake of interventions is encouraging for the goal of evaluating the process and outcomes of multiple early life interventions using the innovative interventional cohort approach. BiBBS covers a period before, during and after the coronavirus disease 2019 (COVID-19) pandemic which adds scientific value to the cohort.

## Introduction

The first 1,001 days, from conception to a child’s 2 ^nd^ birthday, are recognised as the most critical time to intervene and reduce or prevent the impact of negative exposures on a child’s development ^1, 2^. However, despite growing evidence of the importance of early prevention and intervention, there is a paucity of interventions available during this developmental period that have high quality evidence of effectiveness, and even fewer with evidence of reducing inequalities in early years outcomes ^3– 5^.

Randomised controlled trials (RCTs) have long been hailed as the gold standard for clinical research; however, there is growing recognition that the reliance on RCTs to determine effectiveness is not always ideal for public health interventions that are delivered in practice ^6^. For many interventions, randomisation is neither feasible nor ethical. Where randomisation is feasible, traditional RCTs are expensive to deliver and may lack ecological validity, especially where there are complex structural and social contexts to be considered within an evaluation ^6^. In addition, particularly for early life interventions ^7^, effects may take years to realise and RCTs don’t always have the capacity or ability to conduct long-term follow-up. To address these concerns, the Born in Bradford’s Better Start (BiBBS) interventional birth cohort study was established in 2016 with the primary aim of providing an innovative and efficient cohort platform for evaluations of multiple early life interventions ^4^.

The interventions for evaluation within BiBBS include those that are delivered through Better Start Bradford, a National Lottery Community Fund programme ^8^ which works in three ethnically diverse and deprived areas of the city. The aim of the ‘A Better Start programme’ is to give children the best start in life, using preventative interventions to support socio-emotional development, language and communication development, and nutrition, in 0–3 year olds. Interventions include a range of parenting programmes, one to one peer-support, and enhanced clinical care ^8^. Given the high vulnerability of the population, the majority of interventions are offered universally within the area, however, some interventions target a particular vulnerability (e.g. mild symptoms of depression or a high body mass index) and therefore have some eligibility criteria which women must meet to be accepted. Details of all interventions and any eligibility restrictions can be seen on the Better Start Bradford website ^8^. Given the paucity of evidence-based interventions in the early years at the time of set-up ^3^, the majority of interventions selected were science-based (i.e., based on theory of what works) rather than evidence-based.

The interventional cohort approach is a novel design that aims to support multiple efficient, cost-effective and timely implementation and effectiveness evaluations. The cohort life course approach enables longer-term follow-up of outcomes, and consideration of the wider complex context within which interventions are delivered. Planned methods for evaluation using the BiBBS cohort include pragmatic RCTs including trials within cohorts (TWiCs) and quasi-experimental designs (QEDs) such as propensity score matching and regression discontinuity models ^4^. Randomisation into interventions is only undertaken where it is ethical and feasible to do so, for example, if there is more demand than capacity for a universal intervention. Participants consent to be randomised as a part of the recruitment process into BiBBS, more details can be seen in the study protocol ^4^. Where randomisation is neither ethical or feasible, QEDs are used. The value of this approach as an alternative to traditional RCTs is yet to be fully demonstrated and as the first in a growing number of interventional cohorts being implemented internationally ^9, 10^, we are keen to share unique learning about the feasibility and potential of this approach.

The aims of this paper are, therefore to test the feasibility and value of this novel method by:

a) providing a description of the cohort population to promote BiBBS as a leading cohort representing seldom heard and vulnerable populations;

b) exploring the feasibility of the innovative interventional cohort method as a platform to undertake multiple effectiveness evaluations.

The objectives of the paper are to describe:

- the recruitment and reach of the cohort;
- the key characteristics of the cohort participants;
- the uptake of the Better Start Bradford interventions across the cohort;
- the feasibility of BiBBS to complete effectiveness evaluations of the Better Start Bradford interventions, and to share key challenges.

## Methods

### Setting

Bradford, based in the North of England, is the 6 ^th^ largest metropolitan district in England. It has a young population with high levels of deprivation and ethnic diversity. In the three inner-city areas where the Better Start Bradford interventions are offered (Bowling and Barkerend, Bradford Moor and Little Horton), the majority of families are of Pakistani heritage and live in the most deprived decile of deprivation in relation to England and Wales, as assessed by the Index of Multiple Deprivation (IMD) ^4^.

### Recruitment process

Recruitment to the BiBBS cohort began in January 2016, with a planned completion date of December 2023 and a target sample size of 5,000 pregnancies. Recruitment processes are described in full in the BiBBS protocol paper ^4^, and the full study protocol can be seen at: https://www.protocols.io/view/born-in-bradford-s-better-start-an-experimental-bi-cgrhtv36. To date, the main recruitment process has taken place within the Glucose Tolerance Test clinic, (which until 2020, was universal in the local hospital) at ~26 weeks of pregnancy, or in the community during pregnancy or up to two weeks postpartum. Where possible, women have been recruited in their preferred language either by bilingual researchers or using the maternity interpreting services, and invited to: complete an in-depth baseline questionnaire on their family health, wellbeing and social circumstances; provide biological samples (blood and urine in pregnancy, cord blood at birth and a hair sample after birth); consent to linkage of their and their child’s routinely collected health and education data, and data relating to engagement in Better Start Bradford interventions. The consent process is staged, so that mother’s first consent to routine data linkage as this is essential to be a part of the cohort study. They then choose to consent to complete the baseline questionnaire (which is encouraged but not mandated) and to biological samples and future contact which are optional.

### Eligibility

Eligibility for the BiBBS cohort requires women to have: registered to give birth at the local hospital (Bradford Royal Infirmary, Bradford Teaching Hospitals NHS Foundation Trust); reside within the Better Start Bradford areas (defined by full UK postcode) at the time of approach by the research team; and consent during pregnancy (or up to two weeks postpartum). Each pregnancy a woman has during the time period of recruitment is eligible for cohort participation. All babies born to women from the pregnancy during which they have consented are included in the cohort, i.e., multiple births. Women are excluded if they plan to move out of the Better Start Bradford areas before the birth.

### Interim cohort sample

Women recruited between 1 ^st^ January 2016 and 30 ^th^ November 2019 and who had an estimated due date between the 1 ^st^ of April 2016 and the 8 ^th^ of March 2020, and all babies born of those pregnancies, were included in this interim analysis. This means that all babies in this interim profile were born before the Covid-19 pandemic. As cohort recruitment is ongoing, a number of women who were pregnant on the 30 ^th^ November 2019 were still eligible to be approached and consented into the study; for the purposes of this analysis they are defined as ‘not recruited’, giving an under-estimate of overall recruitment to this date. 

### The Eligible population

To understand how representative the recruited sample are compared to the eligible population, anonymised screening data for all women who had an estimated due date of 1 ^st^ April 2016 to 8 ^th^ March 2020 were shared with the research team. Information on: ethnicity (defined using the categories available in the maternity data); spoken English language ability (determined by the midwife); deprivation (IMD); and time of presentation of pregnancy, were taken from the women’s first appointment (booking) in midwifery health records.

### Baseline questionnaire

Key epidemiological data were collected using an in-depth questionnaire completed at the point of recruitment (‘baseline’) available as *Extended data* ^11^.:. The content of the baseline questionnaire includes multiple validated questionnaires detailed in the study protocol paper ^4^ and has been revised over time based on community and research feedback, with some variables being removed and others added, whilst ensuring the validity of questionnaires is maintained. This is reflected in the proportions of missing data for each variable ^12^. Key domains included in the questionnaire include socio-demographic circumstances, financial and food insecurity, physical and mental health and wellbeing, language, home and neighbourhood environment and nutrition. Ethnicity was defined by the Office for National Statistics census 2011 categories ^13^, deprivation was classified using IMD deciles ^14^; and English language ability was self-reported by the participant.

### Data linkage

The cohort design supports the tracking of families’ participation in, and engagement with, the Better Start Bradford interventions. It also provides the opportunity for long-term follow up using routine health and education data for key developmental outcomes.

Linkage to routinely collected health data was requested from general practice (GP), midwifery and health visiting services located within the Bradford district. Health records were extracted where the NHS number, surname, date of birth and sex match a cohort participant record. Participant address data captured from the GP record was updated monthly to support study administration and enable analysis in relation to residential location over time.

**Figure 1.**
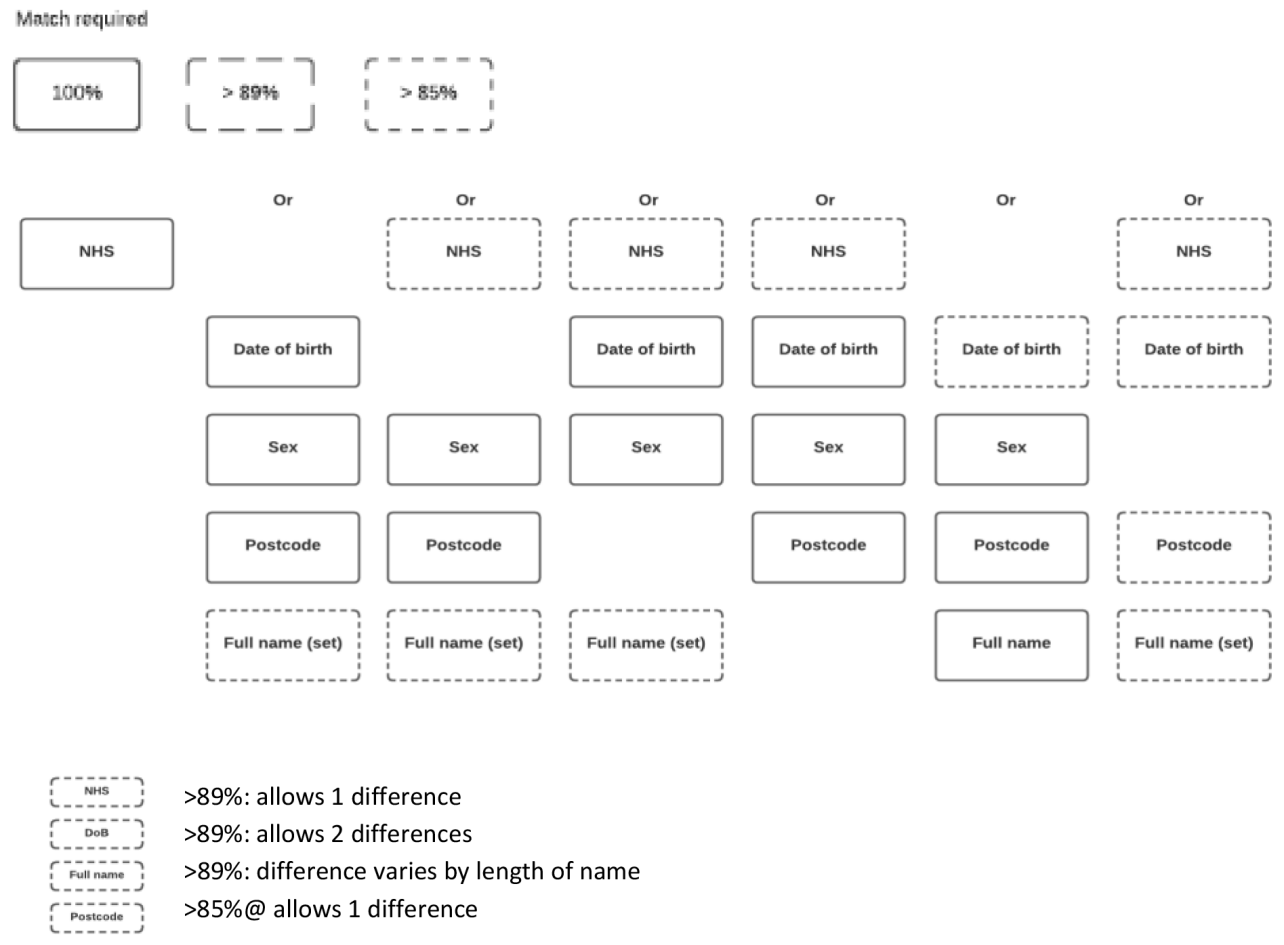
Iterative approach applied to link cohort and intervention data using approximate string matching.

Key to the success of the interventional cohort is linkage to routinely collected information on participation in early life interventions. Where unique identifiers (such as the NHS number) were available, data were extracted and linked as described above for health records. However, for many interventions delivered outside of healthcare settings, collection of unique identifiers was not possible. In these cases, intervention providers were asked to collect full name, date of birth, sex and postcode for all participants to enable probabilistic matching of intervention data to BiBBS data. A number of algorithms were created in Python (version 3.7.1 ^15^) and tested using an iterative approach to linkage. These algorithms utilised the same variables as per the health record linkage (NHS number, surname, date of birth and sex, plus postcode) in varying combinations, and also applied ‘approximate string matching’ to accommodate inaccuracies and missingness in the data (for example, allowing 1 different number within a date of birth). A sample of 100 records from the output of each algorithm was manually reviewed to determine the number of possible false matches. More details of the approach including the packages is available as supplementary data ^11^. The algorithm selected for use within the interventional cohort is shown in Figure 1. This figure shows the variety of matches that could be made including 100% matches, or a small difference between the BiBBS and an intervention record (e.g. an NHS number could have 1 number different = >89% match). This algorithm was selected as it provided the optimal balance between the number of groups generated and the number of possible false matches within groups: false matches were largely limited to records relating to twin children with similar names which could be verified by looking at BiBBS baseline records.

### Data analysis

**Figure 2.**
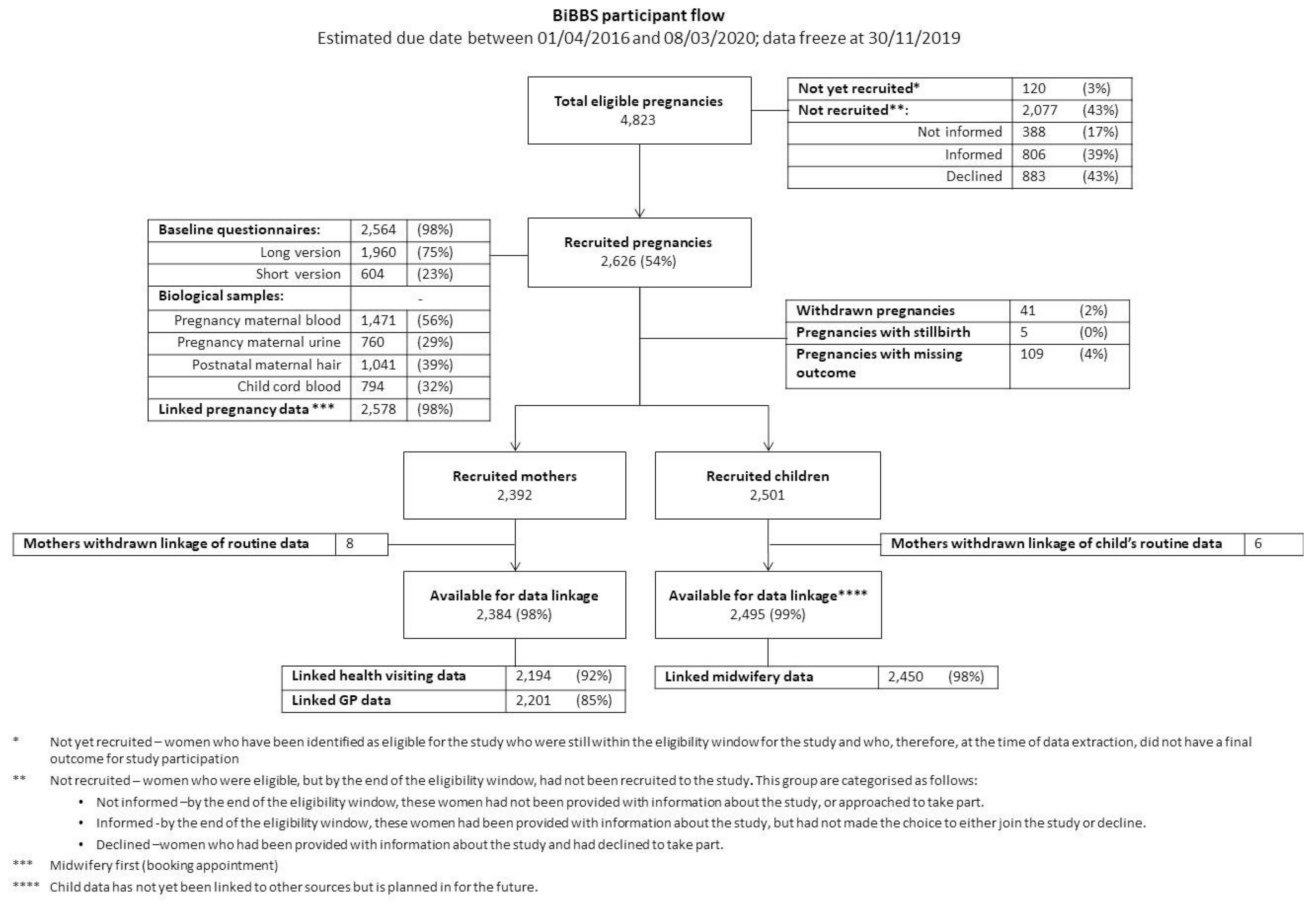
CONSORT Diagram for recruitment into BiBBS.

All data were analysed using descriptive statistics in Stata (version 17, StataCorp, 2021 ^16^). Descriptive statistics of anonymised screening data taken from the women’s first appointment (booking) in midwifery health records were used to compare all eligible pregnancies to those pregnancies that were recruited into the cohort (see Figure 2). Data are described and analysed at the level of the pregnancy.

Baseline questionnaire data are also described at the level of the pregnancy. Different versions of the questionnaire (per Figure 2) were harmonised and merged into a single dataset and numbers and percentages were used to describe the sample. The denominator used for each item is noted in the results tables and varied depending on the number of responses to a particular question; the number of “don’t know” and missing responses are provided for reference. 

All data were analysed as collected except for the following variables which were constructed from the collected data:

For the PHQ-8 ^17^ and GAD-7 ^18^ perinatal mental health measures, a categorical variable was constructed based on the standard clinical scoring classifications for depression (0 to 4 – no depression, 5 to 9 – mild depression, 10 to 14 – moderate depression, 15 to 19 - moderately severe depression, 20 to 24 - severe depression) and anxiety (0 to 4 – no anxiety, 5 to 9 – mild anxiety, 10 to 14 – moderate anxiety, 15 to 21 - severe anxiety ^17, 18^). Moderate, moderately severe and severe categories were collapsed to indicate clinically important symptoms of depression and anxiety. For the Short Warwick-Edinburgh Mental Wellbeing Scale (SWEMWBS) measure ^19^, scores were used to create a categorical variable in order to divide the population into three groups for the purposes of reporting wellbeing: 7 to 19 – low mental wellbeing, 20 to 27 – average mental wellbeing, 28 to 35 – high mental wellbeing. 

Data linkage to routine health data from maternity records is described and analysed at the level of the pregnancy. All other data linkage to routine health data and to Better Start Bradford interventions is described and analysed at the level of the mother.

To explore intervention participation, two levels of intervention exposure were constructed: enrolment (the individual registered to take part in an intervention) and participation (the individual received at least one substantive contact). Completion of the intervention was not considered here because the interim nature of this analysis meant that many participants were still participating in interventions, thereby making completion data incomplete and potentially misleading. Similarly, a number of BiBBS participants’ children are not yet old enough to be eligible for interventions aimed at toddler and pre-school aged children. As such, the enrolment figures were only reported for perinatal interventions where enrolment was during pregnancy or the first year after birth. For exploratory analyses of the conversion rate from enrolment to participation, all interventions were included. The mother’s first pregnancy and their first enrolment in each project were used for this analysis.

To determine feasibility of each intervention for an effectiveness evaluation within BiBBS, the following criteria were used: successful implementation, a sufficient sample size to detect an effect size, an available control group, suitability for an RCT or QED, and outcomes available in routinely collected data.

### Ethical approval

The protocol for recruitment and collection of baseline and routine outcome data and biological samples for the cohort has been approved by Bradford Leeds NHS Research Ethics Committee (15/YH/0455). Research governance approval has been provided from Bradford Teaching Hospitals NHS Foundation Trust.

## Results

### Recruitment and data availability

Figure 2 provides the CONSORT diagram for cohort recruitment and availability of data. Of 4,823 eligible pregnancies, 2,626 (54%) pregnancies were recruited between January 2016 and November 2019 and had an expected due date between 1 ^st^ April 2016 and 8 ^th^ March 2020. Baseline questionnaires were completed for 2,564 (98%) of recruited pregnancies. These pregnancies resulted in 2,501 children in the cohort and 2,392 mothers in the cohort. Routine data linkage was completed for 2,384 mothers and 2,495 children.

### Study reach and representativeness

Table 1 compares participant characteristics of the recruited BiBBS population (pregnancies) to the eligible pregnant population (pregnancies) living in the Better Start Bradford areas. The BiBBS population is representative of the key characteristics of the eligible pregnant population.

**Table 1.** Comparison of the eligible pregnant Better Start Bradford population to the recruited BiBBS pregnant population, by variables of interest.

	Eligible population ** (n=4,703 pregnancies)	Recruited population (n=2,626 pregnancies)
**Age group (years)**		
Less than 20	248 (5%)	102 (4%)
20–24	984 (21%)	542 (21%)
25–29	1,477 (31%)	851 (32%)
30–34	1,212 (26%)	698 (27%)
35 and over	690 (15%)	359 (14%)
Age not known	92 (2%)	74 (3%)
**Parity**		
0	1,345 (29%)	823 (31%)
1	920 (20%)	524 (20%)
2	741 (16%)	409 (16%)
3	436 (9%)	231 (9%)
4 or more	373 (8%)	148 (6%)
Parity not known	888 (19%)	491 (19%)
**Ethnicity**		
Pakistani	2,340 (50%)	1,428 (54%)
White British	574 (12%)	285 (11%)
White Other	427 (9%)	159 (6%)
Other	856 (18%)	455 (17%)
Ethnicity not known	506 (11%)	299 (11%)
**IMD decile**		
1	3,758 (80%)	2,071 (79%)
2	757 (16%)	441 (17%)
3	51 (1%)	33 (1%)
IMD decile not known	137 (3%)	81 (3%)
**Understanding of English language**		
Fluent	3,235 (69%)	1,922 (73%)
Some understanding	972 (21%)	492 (19%)
No understanding	359 (8%)	133 (5%)
Level of understanding not known	137 (3%)	79 (3%)
**Gestation at booking ^a^**		
12 weeks or less	3,578 (76%)	2,134 (81%)
13-20 weeks	669 (14%)	303 (12%)
More than 20 weeks	332 (7%)	105 (4%)
Gestation not known	124 (3%)	84 (3%)

^a ^This indicates the gestation of the pregnancy at the time the woman first presented at Bradford Teaching Hospitals NHS Foundation Trust. ** where the recruitment outcome is known (n=4,703; excluding pregnancies ‘Not yet recruited’).

### Key characteristics of BiBBS participants

Table 2 shows that the BiBBS cohort has recruited a population of high ethnic diversity: 1,571 (61%) of pregnancies are from women of Pakistani heritage; 296 (12%) White British; 213 (8%) other South Asian heritage; 150 (6%) were of White Polish, Czech, Slovakian or Roma ethnicities. This diversity brings with it a large sample of pregnancies of women born outside of the UK (n=1,382, 54%) and with difficulty understanding English (n=530 34%). The majority of participants (2,145, 84%) live in the lowest decile of IMD.

**Table 2.** Demographic characteristics of BiBBS participants from the baseline questionnaire (n = 2,564 pregnancies).

	N (%)
**Ethnicity (n=2,546)**	
***White***	
White British	296 (12%)
White Polish/ Slovakian/ Romanian/ Czech	150 (6%)
Other White	58 (2%)
***South Asian***	
British Pakistani/Pakistani	1,571 (61%)
British Indian/Indian	70 (2%)
British Bangladeshi/ Bangladeshi	143 (6%)
***Black***	
Black Caribbean	7 (<1%)
Black African	85 (3%)
***Mixed heritage***	48 (3%)
Other	115 (5%)
Do not wish to answer	4
Missing	10
**Migrant to the UK (n=2,555)**	
Yes	1,382 (54%)
No	1,173 (46%)
Missing	9
English Language Ability * (n=1,510)	
***Understand what people say in English***	
Not at all/ a little	322 (20%)
Some	208 (14%)
Quite well	315 (21%)
Very well	665 (44%)
Missing	0
**IMD Decile 2019 (n=2,551)**	
Most deprived	2,145 (84%)
Second most deprived	401 (16%)
>Second most deprived	5 (<1%)
Missing	13

*Asked those for whom English was not their first language.

Table 3 shows the social and living circumstances of the BiBBS participants. The majority of participants are married (n=2,010, 79%). Almost one-third (n=805, 32%) were related to the father of their baby, and of these, 462 (58%) were first cousins. The majority of participants and/or their partners were employed; however, 569 (23%) reported financial insecurity (just about getting by or finding it difficult to manage) and 275 (15%) reported food insecurity (reporting that they often or sometimes did not have enough food and had no money to buy more). 314 (13%) reported having no or little social support (0 or 1 other person to rely on).

**Table 3.** Living, social and financial circumstances (n=2,564 pregnancies).

**Relationship with the baby’s natural father (n=2,551)**	
Married	2,010 (79%)
In relationship but not married	380 (15%)
Separated or divorced	132 (5%)
Other	29 (1%)
Missing	13
**Are you related to the baby’s natural father? (n=2,529)**	
Yes	805 (32%)
No	1,724 (68%)
Missing	13
**If you are related to the baby’s father, how are you ** **related? (n=798)**	
First cousin	462 (58%)
Second cousin	153 (19%)
Other related by blood	183 (23%)
Don’t know	7
Missing	0
**Participant currently employed (n=2,550)**	
Yes	870 (34%)
No	1,680 (66%)
Missing	14
**Participants’ partner currently employed (n=2,390)**	
Yes	2,056 (86%)
No	334 (14%)
Don’t know	19
Missing	155
**Highest qualification (n=2,419)**	
No qualifications	225 (9%)
5 or less GCSE (grades A-C) or equivalent	776 (32%)
5 or more GCSE (grades A-C) or equivalent	306 (13%)
A levels or equivalent	295 (12%)
Degree or equivalent	766 (32%)
Other	14 (<1%)
Don’t know	51
Missing	131
**Financial Security (n=2,441)**	
Living comfortably	877 (36%)
Doing alright	995 (41%)
Just about getting by	398 (16%)
Finding it quite/very difficult	171 (7%)
Do not wish to answer	77
Don’t know	30
Missing	16
**Food insecurity (n=1,871)**	
Not having food that lasts and having no money to buy more	
Never true	1,596 (85%)
Sometimes true	228 (12%)
Often true	47 (3%)
Do not wish to answer	70
Missing	19
Cut the size of meals/eat less/skip meals because there was not any food (n=1,884)	
No	1,772 (94%)
Yes	112 (6%)
Do not wish to answer	56
Missing	624
**Overcrowding (n=1,999)**	
Less than 2 people per bedroom	1,570 (79%)
2 or more people per bedroom	399 (20%)
Missing	30
**Social Support: How many people can you count on in ** **times of need? (n=2,505)**	
0-1	314 (13%)
2-5	1,170 (47%)
6-9	361 (14%)
10 or more	660 (26%)
Don’t know	41
Missing	18
**How many of the people you can count on are from your ** **neighbourhood? (n=1,936)**	
None	338 (17%)
Some	480 (25%)
Most	300 (15%)
All	818 (42%)
Missing	24
**Number of residential moves in the past 5 years (n=1,784)**	
0	603 (34%)
1	782 (44%)
2	198 (11%)
3+	201 (11%)
Missing	176
Child residential mobility by age 4 years (n= 717 *)	
Still living in Better Start Bradford area	554 (77%)
Still living in Bradford local authority area	670 (93%)

*Total number of children aged 4+.

Overall, 399 (20%) participants were living in overcrowded housing (defined as 2 or more people per bedroom) and 399 (22%) had moved two or more times in the past five years. Using child GP records, high levels of residential mobility were found for the cohort children: by the age of four 189 (27%) had moved once and 104 (15%) had moved two or more times. The majority of children remained within the Bradford District area (n=539, 93%), with 554 (77%) still residing in the Better Start Bradford area by the age of 4.

Table 4 shows the physical and mental health of participants during pregnancy. A large number of respondents reported symptoms of depression: 749 (31%) mild symptoms and 351 (15%) moderate/severe symptoms of clinical importance. 367 (20%) reported mild anxiety symptoms and 190 (10%) moderate/severe symptoms of clinical importance. Over half of participants were defined as being overweight (772 (30%)) or having obesity (642 (26%)) during pregnancy. 272 (11%) of participants reported smoking at the time of their first midwife appointment.

**Table 4.** Physical and mental health during pregnancy (n=2,564 pregnancies).

**Depressive Symptoms (PHQ-8) (n=2,386)**	
0 – 4 (None)	1,286 (54%)
5 – 9 (Mild)	749 (31%)
10 – 27 (Moderate/Severe)	351 (15%)
Missing	178
**Anxiety Symptoms (GAD-7) (n=1,845)**	
0 – 4 (None)	1,288 (69%)
5 – 9 (Mild)	367 (20%)
10 – 24 (Moderate/Severe)	190 (10%)
Missing	115
**Wellbeing (SWEMWBS) (n=2,101)**	
7 – 19 (Low Wellbeing)	178 (8%)
20 – 27 (Average)	724 (34%)
28 –35 (High)	1,199 (57%)
Missing	463
**Self-Reported Physical Health (n= 2,533)**	
Good - Excellent	1,947 (77%)
Fair	476 (19%)
Poor	110 (4%)
Do not wish to answer	7
Don’t know	6
Missing	18
**BMI at first midwife appointment (n = 2,578)**	
Underweight (<18.5)	123 (5%)
Healthy weight (18.5 – 24.9)	973 (38%)
Overweight (25 – 29.9)	772 (30%)
Obese (30 – 39.9)	584 (23%)
Severely obese (40+)	58 (3%)
Missing	68
**Self-reported smoking at first midwife appointment ** **(n = 2,573)**	
Current smoker	272 (11%)
Ex-smoker	182 (7%)
Never smoked	2,086 (81%)
Unknown	5
Missing	33

Table 5 shows the birth outcomes available for this sample. 211 (9%) of babies had a low birth weight, and 431 (17%) were small for gestational age. Although 2,075 (81%) participants reported in the baseline survey that they intended to “at least give breastfeeding a try”, 1,430 (68%) gave breast milk as their baby’s first feed, and on discharge from hospital 1,179 (52%) of mothers were breastfeeding, 378 (17%) were partially breastfeeding and 721 (32%) were bottle feeding.

**Table 5.** Birth outcomes (n=2,578 pregnancies).

**Sex (n=2,502)**	
Male	1,248 (50%)
Female	1,253 (50%)
**Mode of delivery (n=2,503)**	
Vaginal	1,922 (77%)
Caesarean	581 (23%)
**Preterm birth (n=2,365)**	
<259 days	168 (7%)
**Birth weight by gestational age (n=2,501)**	
Small	431 (17%)
Normal	1,821 (72%)
Large	225 (9%)
Birth weight * (n = 2,450)	
High (>4500g)	197 (8%)
Normal (2500–4499g)	2,045 (83%)
Low (1000–2499g)	174 (7%)
Very low (<999g)	37 (2%)
**Birthweight percentile (n=2,477)**	
<25	885 (36%)
26–50	601 (24%)
51–75	505 (20%)
>75	486 (20%)

*WHO, 2015.

### The uptake of the Better Start Bradford interventions

**Figure 3.**
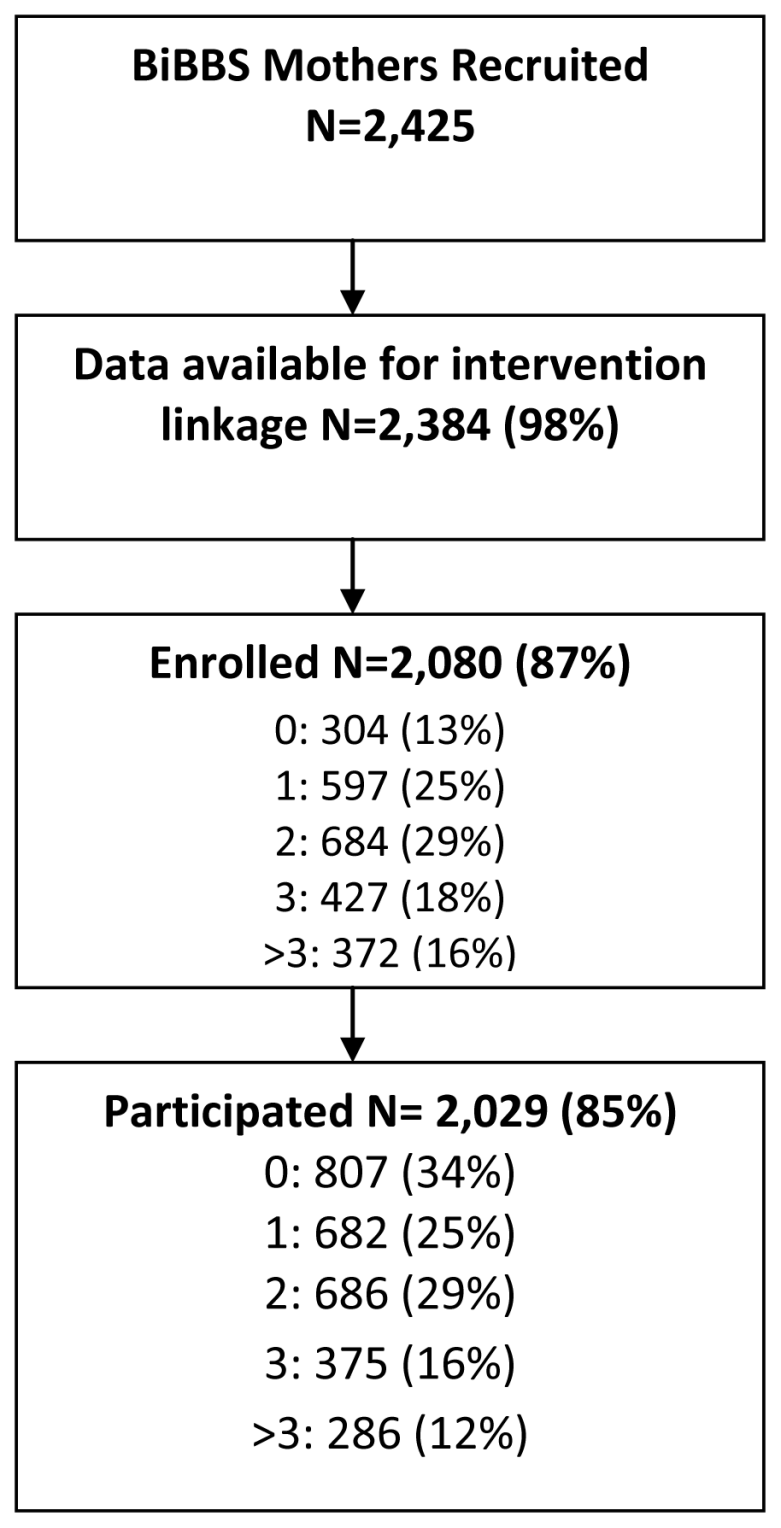
The number ofinterventions across the Better Start Bradford programme that BiBBS mothers had engaged in.

Figure 3 shows the number of interventions across the Better Start Bradford programme that BiBBS mothers had engaged in: 2,080 (87%) of mothers had enrolled (registered to take part in an intervention) onto one or more interventions and 2,029 (85%) of mothers participated (received at least one substantive contact) in one or more interventions.

The total number of BiBBS mothers who took part in a perinatal intervention varied from 61 to 1,491 ( Table 6). The percentage of mothers who enrolled and then participated in the interventions varied from 48% to 100%. Table 7 shows variation in participation by the characteristics of the interventions. For example, interventions that require active participation (e.g., having to proactively enrol / attend a session) had an 80% participation rate compared to 99% in interventions that require passive participation (e.g., enrolled as a part of standard practice / no active attendance). Similarly, interventions that required a short-term commitment (2–6 weeks) had higher levels of participation (91%) than those that required a longer-term (>12 weeks) commitment (78%). 

**Table 6.** Number of BiBBS mothers enrolled and participated in interventions, and key characteristics of the interventions.

Intervention	Enrolled	Participated	Participated/ Enrolled %	Participation Type	Universal/ Targeted	Place of Delivery*	Group / Individual	Commitment+
**Perinatal**								
Baby Steps	117	85	73%	Active	Targeted	Mixed	Group	Long fixed
HAPPY	61	47	77 %	Active	Targeted	Community	Group	Long fixed
Family Action	149	140	94%	Active	Targeted	Home	Individual	Variable
Breastfeeding Support	519	251	48 %	Active	Universal	Home	Individual	Variable
Continuity of Carer Midwifery	878	878	100	Passive	Universal	Community	Individual	Long fixed
Better Start Imagine	1,492	1,492	100	Passive	Universal	Home	Individual	Variable
PERINATAL TOTAL	3,216	2,893	90%					
**Toddler/Pre-School**								
Cooking for a Better Start	44	43	98%	Active	Universal	Community	Group	Short fixed
HENRY	48	47	100%	Active	Universal	Community	Group	Medium fixed
Incredible Years Toddler	88	75	85%	Active	Universal	Community	Group	Long fixed
Talking Together – Screening	942	841	89%	Active	Universal	Home	Individual	Short fixed
Talking Together – Intervention	402	365	91%	Active	Targeted	Home	Individual	Short fixed
Forest Schools	77	64	83%	Passive	Universal	Community	Group	Medium fixed
TODDLER/PRE-SCHOOL TOTAL	1,601	1,435	90%					

**Table 7.** Mother’s enrolment and participation by intervention characteristics.

Intervention Characteristics	Type	Enrolments	Participations	Participated/ enrolled (%)
**Participation**	Active	2,370	1,894	80%
	Passive	2,447	2,434	99%
**Targeting**	Universal	4,088	3,691	90%
	Targeted	729	637	87%
**Delivery**	Home	3,504	3,089	88%
	Mixed	130	98	75%
	Outside home	1,183	1,141	96%
**Group/Individual**	Individual	4,395	3,980	91%
	Mixed	117	85	73%
	Group	305	263	86%
**Stage**	Perinatal	3,216	2,893	90%
	Childhood	1,601	1,435	90%
	Perinatal	266	225	85%
	Pregnancy	939	925	99%
**Commitment level**	Choice	2,160	1,883	87%
	Long-term fixed	266	207	78%
	Short-term fixed	446	408	91%
	Medium-term fixed	125	111	89%
**Theme**	Nutrition	672	388	58%
	Language	2,836	2,698	95%
	Socio-emotional	1,309	1,242	95%
**Total**		**4,817**	**4,328**	

### Feasibility of BiBBS to Complete Effectiveness Evaluations

The purpose of the BiBBS cohort was to be able to enhance the evidence base of early years interventions by carrying out multiple evaluations of interventions being delivered within practice. Table 8 shows the feasibility and progress of effectiveness evaluations for each intervention based on: evidence of successful implementation; a sufficient sample size to detect an effect size; an available control group; suitability for a RCT or QED; and outcomes available in routinely collected data. At the point of analysis, a number of interventions had progressed to the stage of effectiveness evaluations, these included: a pragmatic RCT of the continuity of carer midwifery model using randomisation at point of care and data from women in BiBBS; a TWiCs feasibility evaluation of Incredible Years Toddler alongside a larger QED; QED evaluations of Baby Steps and HENRY (with controls matched to intervention participants within BiBBS using propensity score matching). A feasibility RCT of the Talking Together intervention using a wait-list control was completed in 2021 and demonstrated the feasibility for a full RCT and evidence of promise on children’s vocabulary and the warmth of the parent-child interactions ^20^. Further evaluation of this intervention requires a larger sample size than is available within the remaining timeframe of the Better Start Bradford programme, but is planned to be undertaken when wider roll-out of the intervention is underway. Other interventions have been found to be not suitable for an effectiveness evaluation, for example, a feasibility TWiCs of the HAPPY intervention (a perinatal parenting and healthy eating programme targeting overweight/obese women) was not able to be completed as insufficient women engaged in the intervention, leading to the de-commissioning of this intervention. Further information on these interventions can be found at www.betterstartbradford.org.uk ^8^.

**Table 8.** Assessment of the feasibility for effectiveness evaluation of each intervention.

PROJECT	No. Participated	Evidence of good implementation?	Sufficient sample size ^#^	Control Sample?	Primary Outcome	Available in Routine Data?	Evaluation Planned
**Evaluation Completed**							
Talking Together – Intervention	365 *	Y	N *	n/a	Language Development	N	Feasibility RCT completed
**Evaluation Underway**							
Baby Steps	85	Y	Y	Y	Maternal mental health	N, Proxy	QED using propensity score matching
HENRY	47 *	Y	Y *	Y	Child BMI	Y	QED using propensity score matching
Incredible Years Toddler	75 *	Y	Y *	Y	Child Behaviour	Y	QED using propensity score matching; TWICS pilot
Continuity of Carer Midwifery	878	Y	Y	Y	Birth Outcomes / Maternal mental health	Y	RCT using cohort data
**Potential Evaluation, Scoping underway**							
Breastfeeding Support	251	Y	Y	TBC	Breastfeeding Duration	Y	Scoping QED using propensity score matching
Better Start Imagine	1,492	Y	Y	TBC	Language Development	N, Proxy	Scoping, time series
Forest Schools	64 *	Y	TBC	Y	School Readiness	Y	Scoping QED using cluster (nursery level) matching
**Not suitable for ** **evaluation**							
Cooking for a Better Start	43	Y	N	n/a	n/a	n/a	Not feasible for RCT or QED, capacity of project too small, no routine data outcome identified
Family Action	140	TBC	Y	N	Maternal mental health	N, Proxy	Not ethical for RCT, not feasible for QED as not possible to identify a matched control group
HAPPY	47	N	N	n/a	Child BMI	Y	Pilot TWiCS undertaken, but participation rates too low. Intervention de-commissioned

^#^ The sample size for each intervention is fixed dependant on the project’s capacity, participation and completion rates. This column estimates whether the predicted fixed sample size will be sufficient to detect a significant effect size on the primary outcome for each intervention. *Many BiBBS participants have not yet reached the eligibility age for these interventions, and the sample size will increase in the coming years. A sufficient sample size in these cases are based on projected sample sizes

## Discussion

BiBBS is an innovative interventional cohort with a contemporary sample of women and children living in marginalised and disadvantaged communities, including a large proportion of ethnic minorities, migrants, those with limited English language ability and those living in deprivation. BiBBS achieved an overall recruitment rate of 54%, and has successfully recruited a sample who are representative of the eligible pregnant population. The diverse sample is a key strength and unique element of the BiBBS cohort, enabling insights into the socio-economic and developmental outcomes of communities who are all too often under-represented in research studies.

Considerable investment was made at the start of the cohort in building deep-rooted community trust and engagement including consultation on all recruitment processes; dissemination events; recruitment within maternity clinics; a research team representative of the community, including bilingual researchers, and access to a pool of clinical interpreting services. Plans are in place to examine patterns of recruitment by key demographic factors in order to help understand how such factors affect recruitment to studies like this, as well as to examine how the recruitment strategies adopted by our study may have influenced the participation (or non-participation) of different groups. 

The baseline questionnaire data and routine linked data highlight the varied experiences of families with many reporting good health and wellbeing and financial security, whilst others report multiple vulnerabilities that may impact upon the health, wellbeing and development of their children. These include high maternal obesity, poor maternal mental health, financial insecurity, overcrowding, low levels of breastfeeding and high residential mobility, all of which need to be addressed to reduce inequalities in child outcomes.

BiBBS covers a period before, during and after UK austerity measures, the coronavirus disease 2019 (COVID-19) pandemic, and the cost of living crisis which have all hit families hard. This adds scientific value to the cohort, enabling researchers to describe changes in circumstances over time. The population described in this paper, all of whom had babies born before the 23 ^rd^ March 2020, when the first COVID-19 restrictions were enforced in the UK, fortuitously acted as a pre-pandemic baseline for our research into the impacts of the pandemic on key outcomes such as mental health and financial and food insecurity ^21^. Women in BiBBS who were pregnant / gave birth during the COVID-19 pandemic are now a key population for our research to understand the experiences of being pregnant and growing up during the pandemic ^22^.

In addition, the cohort provides the opportunity to compare the socio-economic, health, wellbeing and development of BiBBS families with families in previous cohorts (describing changes over time) including the BiB family cohort (2007–2011) ^23^ living in the same areas of Bradford, particularly around key cultural and societal outcomes such as levels of consanguinity.

The biobank samples arising from BiBBS will also be of value in the future to demonstrate important linkages between biomarkers and genome data and social and health outcomes. In addition to blood and urine samples, BiBBS has collected hair samples from mothers after birth which can be analysed for levels of cortisol stress hormone in the three trimesters of pregnancy.

### Evaluation of early life interventions

A high proportion of BiBBS families engaged in one or more of the interventions aimed to improve outcomes, and reduce inequalities, in the early years. However, levels of enrolment and participation varied between interventions, and exploratory analysis indicates that this may in part be determined by the characteristics of the interventions such as the requirement for active/passive participation and the length of commitment to a programme. This variance merits further investigation as there may be important learning from these data as to which types of interventions should be delivered to ensure acceptability and engagement within communities. Such an evaluation is planned within the BiBBS team, and the choice of optimal methods with which to do such complex analyses are also underway. It will also be important to assess further the characteristics of those families who do and do not participate in the interventions to ensure that they are reaching the right families in order to reduce inequalities.

The high level of participation in interventions is encouraging for the evaluation of process and outcome measures. Whilst a number of interventions are not suitable for effectiveness evaluations many are, using pragmatic RCT and QEDs. The BiBBS cohort also offers the opportunity to explore how exposure to a number of early years interventions affects child outcomes, and whether different combinations of intervention exposure have differing effects on child outcomes ^24^.

### Challenges for interventional cohort evaluations

Effectiveness evaluations of interventions delivered within routine practice are challenging, and many of the interventions being delivered have been deemed not feasible for effectiveness evaluations because they have either not been well implemented, do not have a sufficient sample size, and/or lack an identifiable control group. The interventions being evaluated here are commissioned through the Better Start Bradford programme and delivered as ‘usual care’ within services. Whilst the research team are able to have some input into how these interventions are designed, (e.g., the selection of control groups, or the collection of outcome data) they are delivered independently. As in all ‘real life’ interventions, this means that where there are issues with implementation (e.g., difficulties recruiting families) or commissioning decisions (e.g., an unexpected end to commissioning, or service re-design), these impact on our ability to utilise the cohort to evaluate the impact of the intervention. To enable the intervention and evaluation to be delivered successfully side by side, we have taken a partnership approach, working closely with commissioners, service providers, stakeholders, the community and researchers at every stage of service design, implementation and evaluation. We have developed a range of practice strategies, tools and templates with our partners to facilitate this ^25, 26^.

BiBBS was designed to make use of life course, routinely collected health and education data to obtain the primary outcomes for the effectiveness evaluations. As we have progressed, we have identified gaps in these data which are relevant nationally, and which make evaluations in practice more challenging, and in some cases, impossible. For example, there are no validated assessments of the mother-child relationship or a universally collected measure of the language development of a child in early years services. Whilst assessment of, and support for, perinatal mental health is a key priority for universal midwifery and health visiting, the data systems do not support documentation of the assessments that are undertaken, with national systems unable to report on the prevalence of perinatal mental health. We have undertaken steps to improve this situation locally, including the assessment of an existing measure of the mother-child relationship ^27, 28^ and the co-production and validation of a new measure for this outcome ^29^, development of a proxy measure for perinatal mental health ^30^, as well as additional data collection within the cohort and use of data collected within the existing interventions.

An additional challenge to our planned evaluations is the finding that, by the age of 4, almost one-quarter of children had left the Better Start Bradford areas. Whilst the majority of these children remain within the City of Bradford, (and so their outcomes can continue to be accessed and linked), a move out of the area means that their exposure to the interventions will be less. This is an impact that requires careful monitoring over the coming years, particularly for interventions that are designed for older, pre-school children.

BiBBS has also provided unanticipated benefits including the promotion of a system-wide research culture, the opportunity to inform the service design of interventions by defining the population needs for interventions and setting feasible participation and completion targets. It has enabled a number of in-depth implementation evaluations ^31^ to be completed which have demonstrated the key successes of many interventions, as well as highlighting a number of interventions which are not feasible for delivery within the Better Start Bradford communities ^32^. 

### Opportunities for collaboration

BiBBS offers opportunities to researchers from across the globe to collaborate with BiBBS to complete further investigations of this population and to use the BiBBS cohort to evaluate potential interventions. Any such collaborations should be initiated by contacting the lead author, and will be subject to review and approval by the BiB Executive Committee.

## Conclusion

The novel approach of the BiBBS interventional cohort has the potential to combine the traditional observational methods used in cohorts to characterise and track the level of need in vulnerable families with real world evaluations to understand the impact of multiple early years interventions on inequalities in child outcomes. This paper highlights the need for new methods to enhance the evidence base, whilst also demonstrating the complexity of evaluations within real world settings. This contributes to the scarcity of high quality evidence for early years interventions. A combination of well accepted RCT designs, complemented by interventional cohorts that embed RCTs and QEDs to evaluate interventions delivered in practice will help to enhance the evidence base in the coming years.

## Data Availability

Researchers are encouraged to make use of the BiBBS data, which are available through a system of managed open access. Before you contact us, please make sure you have read our Guidance for Collaborators. Our BiB Executive reviews proposals on a monthly basis and we will endeavour to respond to your request as soon as possible. You can find out about the different datasets in our Data Dictionary. If you are unsure if we have the data that you need please contact a member of the BiB team ( borninbradford@bthft.nhs.uk). Once you have formulated your request please complete the ‘Expression of Interest’ form available here and send to borninbradford@bthft.nhs.uk. If your request is approved we will ask you to sign a Data Sharing Contract and a Data Sharing Agreement, and if your request involves biological samples we will ask you to complete a material transfer agreement. Harvard Dataverse. Supplementary Files for Born in Bradford’s Better Start (BiBBS) Interventional Birth Cohort Study: Interim Cohort Profile. https://doi.org/10.7910/DVN/ZQIUNC ^11^. This project contains the following extended data: STROBE checklist for Born in Bradford’s Better Start (BiBBS) Interventional Birth Cohort Study: Interim Cohort Profile. BiBBS baseline questionnaire Version 5 for BiBBS Supplemental File 1: Further information on the fuzzy matching process for linking intervention participation data to BiBBS. Data are available under the terms of the Creative Commons Zero "No rights reserved" data waiver (CC0 1.0 Public domain dedication).
