# Supplementary material for: Born in Bradford’s Better Start (BiBBS) interventional birth cohort study: Interim cohort profile
Source: Wellcome Open Res. 2023 Sep 21;7:244. Originally published 2022 Oct 3. [Version 2] doi: 10.12688/wellcomeopenres.18394.2 (PMC10565418; doi:10.12688/wellcomeopenres.18394.2)
Supplement: Peer review history

# Reviewer report: Reviewer response for version 2

**DOI:** 10.21956/wellcomeopenres.22243.r67463

**Published:** 2023-10-10

**Authors:** Joseph Murray

**Affiliations:** 1 Federal University of Pelotas, Pelotas, Brazil

The revisions are all helpful and adequate.

 I suggest to clarify in the new Methods section on “Eligible Population” that these data refer to women who reside within the Better Start Bradford areas.

Is the work clearly and accurately presented and does it cite the current literature?

Partly

If applicable, is the statistical analysis and its interpretation appropriate?

Not applicable

Are all the source data underlying the results available to ensure full reproducibility?

Partly

Is the study design appropriate and is the work technically sound?

Yes

Are the conclusions drawn adequately supported by the results?

Yes

Are sufficient details of methods and analysis provided to allow replication by others?

Yes

Reviewer Expertise:

Professor in Epidemiology Programme working on birth cohorts.

I confirm that I have read this submission and believe that I have an appropriate level of expertise to confirm that it is of an acceptable scientific standard.

# Reviewer report: Reviewer response for version 1

**DOI:** 10.21956/wellcomeopenres.20389.r62883

**Published:** 2023-09-07

**Authors:** Joseph Murray

**Affiliations:** 1 Federal University of Pelotas, Pelotas, Brazil

This paper explains the methodology of the Born in Bradford’s Better Start (BiBBS) study, and describes the baseline sample and rates of participation in the study interventions. BiBBS is a highly innovative, important new cohort study, because of its focus on evaluations of many different interventions implemented in the Bradford’s Better Start project. Previous protocol papers on BiBBS have described its planned methodology. This new paper will provide a very useful reference point about recruitment, and the baseline sample included until March 2020. The paper offers some insight into how this type of unusual cohort (including evaluation of many interventions) can be initiated effectively, and some of the challenges involved. This all makes for a useful contribution to the literature.

 The article is well written, and generally succeeds in clearly explaining the nature of the somewhat complex sample, and the methods involved. Most revisions required are about improving clarity further, and revising apparent inconsistencies in the Tables of results, as follows.

1. I did not understand about the period of recruitment and planned analyses. It seems recruitment is still ongoing, but this article reports on mothers recruited up to 8 ^th^ March 2020. Is it expected that BiBBS will run separate analyses on participants recruited before 8 ^th^ March 2020, hence the importance of reporting here on the sample recruited up to that date? Please make it explicit that this end date (in 2020) was used given the COVID-19 pandemic (currently only implicit). The term “data cut” regarding this time point is unclear in the abstract.
2. Page 3 states that consent to data linkage “is essential” to participation. Does this mean, in the eligibility section, that if someone gave consent to interviews but not to linkage the person was not eligible?
3. Consent being included as an eligibility criterion seems to conflict with presenting data on the “eligible population” (e.g. Table 3) vs. the recruited sample. Presumably this eligible population had not all given consent.
4. Figure 1 needs further information. What are the numbers (>89 etc.) at the bottom?
  1. The supplementary file at https://dataverse.harvard.edu/file.xhtml?fileId=6477871&version=1.0 seems to present this Figure again in a different format.
5. Given the different numbers involved depend on whether pregnancies, mothers, or children are counted (because of the slightly unusual situation of having fewer mothers than pregnancies in a cohort), it would be useful to clarify in the Table titles which is being counted.
6. Please clarify somewhere if eligible mothers in the Better Start Bradford catchment area could choose to participate in all of the interventions provided in that area.
7. Regarding Better Start Bradford, page 3 states the interventions are aimed at providing support for development from 0-4 years, but prior publications of BiBBS, and Better Start Bradford webpage refer to 0-3 years.
8. The paper refers to the conduct of pragmatic RCTs in this cohort. Can some further explanation be given about why and how it was possible to randomise for some evaluations, but not others?
9. Figure 3. Specify that the counts represent the number of interventions being participated in.
10. Table 1 shows 73% of the sample as fluent in English, but Table 2 report that only 44% understand English very well.
11. Some variable descriptions in Tables 3 and 4 present “don’t know” or “do not wish to answer” as responses included in the calculation of prevalence. As an example, the calculation of 14% who reported food insecurity is currently based on a denominator including those who didn’t want to answer the question, rather than those who provided information about whether or not they experienced food insecurity. It would generally be better excluding “don’t know” or “do not wish to answer” from the prevalence calculations.
12. There is an error in “Cut the size of meals/eat less/skip meals”, where the categories (including missing) sum to >100%.
13. There are apparently several inconsistencies in the numbers enrolled/participating in interventions between Tables, for example:
  1. Table 6 shows there were 3,216 enrolments in pregnancy interventions, but Table 7 seems to show 939 enrolments in pregnancy interventions.
  2. Regarding individual interventions Table 6 shows 117 enrolled in Baby Steps while Table 8 shows 85.
14. I suggest you re-order the categories of interventions chronologically in Table 7: pregnancy, perinatal, baby, childhood.
15. As part of considering feasibility, Table 8 shows a column whether sample size is adequate for evaluation. Some information is required about how that was determined.

Is the work clearly and accurately presented and does it cite the current literature?

Partly

If applicable, is the statistical analysis and its interpretation appropriate?

Not applicable

Are all the source data underlying the results available to ensure full reproducibility?

Partly

Is the study design appropriate and is the work technically sound?

Yes

Are the conclusions drawn adequately supported by the results?

Yes

Are sufficient details of methods and analysis provided to allow replication by others?

Yes

Reviewer Expertise:

Professor in Epidemiology Programme working on birth cohorts.

I confirm that I have read this submission and believe that I have an appropriate level of expertise to confirm that it is of an acceptable scientific standard, however I have significant reservations, as outlined above.

# Response

**Published:** 2023-09-15

**Authors:** Josie Dickerson

**Affiliations:** Bradford Hospitals National Health Service Trust, UK

We would like to thank the reviewer for their heplful comments which have added clarity to this paper. We address each of the points from the reviewer below, and confirm that we have submitted an updated version of the paper with the suggested amendments.  

1. I did not understand about the period of recruitment and planned analyses. It seems recruitment is still ongoing, but this article reports on mothers recruited up to 8 ^th^ March 2020. Is it expected that BiBBS will run separate analyses on participants recruited before 8 ^th^ March 2020, hence the importance of reporting here on the sample recruited up to that date? Please make it explicit that this end date (in 2020) was used given the COVID-19 pandemic (currently only implicit). The term “data cut” regarding this time point is unclear in the abstract.
*We don’t intend to use this interim dataset separate to the rest of the cohort in the long-term, but as this is a novel interventional cohort method targeting a ‘seldom heard’ population, and the recruitment to BiBBS is over a long timescale, we wanted to do an interim profile analysis to a) test the feasibility and value of this novel method as soon as we had a reasonable sample size and b) promote the seldom heard population within this cohort and share learning about the needs of this vulnerable population. We have amended the introduction to make this clearer. We have also made it explicit that this cut does coincide with babies born before the beginning of the pandemic in the section ‘Interim Cohort Sample’.* *We’ve also changed the use of the term “data-cut” in the abstract to “analysis”.*
2. Page 3 states that consent to data linkage “is essential” to participation. Does this mean, in the eligibility section, that if someone gave consent to interviews but not to linkage the person was not eligible?
*Yes, this is correct, all of our evaluations are reliant on using routinely linked data so it would not be ethical to recruit someone whose data could not be used as a part of the ongoing evaluations. However, our consent is staged, so a mother’s agreement to allow us to link to their routine data comes first in the process. We have added this explanation into the ‘Recruitment Process’ section.*
3. Consent being included as an eligibility criterion seems to conflict with presenting data on the “eligible population” (e.g. Table 3) vs. the recruited sample. Presumably this eligible population had not all given consent.
*This is an important point which was missing as an explicit section in the methods. We have added a section entitled “The eligible population’ into the methods describing how we accessed anonymised aggregate data from midwifery records to compare our recruited sample to the eligible population.*
4. Figure 1 needs further information. What are the numbers (>89 etc.) at the bottom?
*We’ve amended Figure 1 to add in a figure legend at the top and also added a ‘%’ after each number so that it is clear what the numbers are. In addition there is now more explanation of the algorithm in the text to add further clarity.* **The supplementary file at https://dataverse.harvard.edu/file.xhtml?fileId=6477871&version=1.0 seems to present this Figure again in a different format. *This figure includes the 3 algorithms that were assessed for use, before the final algorithm was selected. We hope that the amended text in the ‘Data Linkage’ section of the methods helps to clarify this difference.*
5. Given the different numbers involved depend on whether pregnancies, mothers, or children are counted (because of the slightly unusual situation of having fewer mothers than pregnancies in a cohort), it would be useful to clarify in the Table titles which is being counted.
*This has now been added to the title of each table*
6. Please clarify somewhere if eligible mothers in the Better Start Bradford catchment area could choose to participate in all of the interventions provided in that area.
*We have added further clarification in the introduction to explain that whilst the majority of interventions are open to all women (i.e. universal), some target a specific vulnerability so have eligibility criteria.*
7. Regarding Better Start Bradford, page 3 states the interventions are aimed at providing support for development from 0-4 years, but prior publications of BiBBS, and Better Start Bradford webpage refer to 0-3 years.
*The eligibility is up until the child’s 4 ^th^ birthday, we have amended this to read 0-3 in the introduction for consistency.*
8. The paper refers to the conduct of pragmatic RCTs in this cohort. Can some further explanation be given about why and how it was possible to randomise for some evaluations, but not others?
*We’ve added more detail into the Introduction to explain how this is possible, and refer back to the study protocol for the full detail of these methods.*
9. Figure 3. Specify that the counts represent the number of interventions being participated in.
   
*The text has been amended to specify this.*
10. Table 1 shows 73% of the sample as fluent in English, but Table 2 report that only 44% understand English very well.
*The data differ because the data in Table 1 comes from midwifery routine data (as it is comparing BiBBS participants to the eligible population) whereas the data in Table 2 comes from the cohort baseline questionnaire. The additional section in the methods entitled “the eligible population” (as per point 2 above) now explains these important differences.*
11. Some variable descriptions in Tables 3 and 4 present “don’t know” or “do not wish to answer” as responses included in the calculation of prevalence. As an example, the calculation of 14% who reported food insecurity is currently based on a denominator including those who didn’t want to answer the question, rather than those who provided information about whether or not they experienced food insecurity. It would generally be better excluding “don’t know” or “do not wish to answer” from the prevalence calculations.
*These responses have now been excluded from the prevalence rates, and the percentages in the associated text have also been amended. *  
12. There is an error in “Cut the size of meals/eat less/skip meals”, where the categories (including missing) sum to >100%.
   Need to delete % assigned to missing.
*This % assigned to missing has been deleted and the categories do now add up to 100%.*
13. There are apparently several inconsistencies in the numbers enrolled/participating in interventions between Tables, for example:
  1. Table 6 shows there were 3,216 enrolments in pregnancy interventions, but Table 7 seems to show 939 enrolments in pregnancy interventions.
*Thank you for noticing this error, the categories and numbers in Table 7 have now been amended to align with those in Table 6. *
  2. Regarding individual interventions Table 6 shows 117 enrolled in Baby Steps while Table 8 shows 85.
*In Table 6 the number who were enrolled is 117 and the number who participated is 85. Table 8 reports the number who participated which is 85.*
14. I suggest you re-order the categories of interventions chronologically in Table 7: pregnancy, perinatal, baby, childhood.
*These categories have now been amended to match those in Table 6 as per point 12 above*, *and are now chronological.*
15. As part of considering feasibility, Table 8 shows a column whether sample size is adequate for evaluation. Some information is required about how that was determined.
*This has been added as a footnote to this table – all sample sizes are fixed and dependent on the intervention capacity and their participation and completion rates. *

# Reviewer report: Reviewer response for version 1

**DOI:** 10.21956/wellcomeopenres.20389.r52774

**Published:** 2022-11-17

**Authors:** Tomas Faresjo

**Affiliations:** 1 Division of Community Medicine, Department of Health, Medicine and Care, Linköping University, Linköping, Sweden

This paper provides an interim analysis of The Born in Bradford’s Better Start (BiBBS) interventional birth cohort. This Born in Bradford’s Better Start (BiBBS) cohort study was designed as an innovative cohort platform for the efficient evaluation of early life interventions and to share learning about the feasibility and value of this method.

1. **Is the work clearly and accurately presented and does it cite the current literature?**
  The paper is clearly presented and well structured. Although in this stage of the project with ongoing data collection, there are only descriptive data of the cohort presented. The reference list covers important contributors to this research field. Some tendencies of self-referring could be seen, but several of the researchers in the research group have published many papers and articles in the field.
2. **Is the study design appropriate and does the work have academic merit?**
  This study is an interventional study of a large sample of around 5,000 pregnancies and children. In this report around half of the planned eligible pregnancies are included, since data is still collected. So, in a way, this report is a half-time report but includes a sufficient number of participants to make an appropriate analysis. What stands out is that the design is based on this is an interventional cohort with the general purpose to strengthen vulnerable pregnant women and newborn children coming from disadvantaged areas in Bradford.
  From an academic view, the generalizability, of analyzing this cohort is limited since the ethnical background in this sample is not representing normal UK areas, almost 61% have Pakistani heritage. But the purpose of the study was not to gain a generalizable sample. On the other hand, data from this cohort could be compared to corresponding data of ethnic groups in other sites.
  As clearly pointed out in the paper the golden standard of study design could be randomized controlled trials (RCT). However, that type of study design is not so applicable to public health research.
  This data collection will have a major potential for a variety of studies with high academic standards. I recommend that the research group take advantage of the data collection done before during and after the COVID-19 pandemic which adds an extra chance to scientifically analyze the public health impact of this extraordinary societal crisis. In prospective studies, there are always some drop-outs over time. I also recommend that the research group, if ethically possible, could merge other available healthcare data from primary care or hospital care. The study is very promising and we could expect a variety of good papers from this research. 
3. **Are sufficient details of methods and analysis provided to allow replication by others?**
  Yes, the basic methods and design could be replicated by others but this data collection and design is very specific for the Born in Bradford project.
4. **If applicable, is the statistical analysis and its interpretation appropriate?**
  At this stage, there are not any major statistical analyses made since the purpose is to demonstrate the feasibility of this cohort for public health interventions and a descriptive approach rather than statistical analysis.
5. **Are all the source data underlying the results available to ensure full reproducibility?**
  Yes, the source data underlying the results are available. The researchers are encouraged to make use of the BBIBs data. Further, the study is open to collaboration with other researchers.
6. **Are the conclusions drawn adequately supported by the results?**
  Yes, the conclusions drawn are adequately supported by the results.

**Approval status**

 APPROVED: None or only minor changes in the manuscript are required. Results are presented accurately and the conclusions are justified and supported by the data.

Is the work clearly and accurately presented and does it cite the current literature?

Yes

If applicable, is the statistical analysis and its interpretation appropriate?

Yes

Are all the source data underlying the results available to ensure full reproducibility?

Yes

Is the study design appropriate and is the work technically sound?

Yes

Are the conclusions drawn adequately supported by the results?

Yes

Are sufficient details of methods and analysis provided to allow replication by others?

Yes

Reviewer Expertise:

Professor in Community Medicine and epidemiology.

I confirm that I have read this submission and believe that I have an appropriate level of expertise to confirm that it is of an acceptable scientific standard.
